# Localization and validation of the “Growing Up With Hearing Loss” tool for Brazilian Portuguese

**DOI:** 10.1590/2317-1782/e20250187en

**Published:** 2026-04-27

**Authors:** Daniele Baptista Nery, Aline Daniela Gomes da Silva Vieira, Anderson Vinícius de Moraes Ortega, Thais Corina Said de Angelo, Adriane Lima Mortari Moret, Natália Barreto Frederigue-Lopes, Déborah Viviane Ferrari, Leila Maria Gumushian Felipini, Regina Tangerino de Souza Jacob

**Affiliations:** 1 Departamento de Fonoaudiologia, Faculdade de Odontologia de Bauru – FOB, Universidade de São Paulo – USP - Bauru (SP), Brasil.; 2 Centro Universitário Sagrado Coração – Unisagrado - Bauru (SP), Brasil.

**Keywords:** Hearing Loss, Auditory Rehabilitation, Cultural Adaptation, Communication, Validation

## Abstract

**Purpose:**

To present the process of localization and validation of the Growing Up with Hearing Loss material into Brazilian Portuguese, aiming to expand its applicability in the national context.

**Methods:**

Methodological study of translation, cultural adaptation, and content validation. After authorization from the Ida Institute, the material was localized into Brazilian Portuguese to ensure semantic, cultural, and technical equivalence. The process included expert review, subtitling of 32 videos, cultural adaptation, and the development of questionnaires applied to children, adolescents, and young adults with hearing loss, as well as their families and speech-language pathologists.

**Results:**

The material was localized as Crescendo com a Perda Auditiva and validated by 42 participants. Three groups achieved agreement rates above 75% on the clarity and appropriateness of the content, while the young adult group achieved 74%, close to the established minimum criterion. Suggestions focused mainly on improving video subtitling. The material showed potential to support life transition management, promote communication skills, and support the development of individuals with hearing loss.

**Conclusion:**

Crescendo com a Perda Auditiva proved suitable for the Brazilian context, with the potential to support auditory rehabilitation and the development of social, cognitive, and communication skills. Adjustments to audiovisual content are recommended to optimize subtitle comprehension.

## INTRODUCTION

The World Health Organization (WHO) estimates that over 1.5 billion people worldwide experience some degree of hearing loss, including 34 million children. The lack of diagnosis and early intervention can affect language development, school performance, and lead to social isolation, especially in childhood and adolescence^(1)^.

During these developmental stages, communicative, social, and educational demands become more complex, requiring greater autonomy and adaptation to new contexts. Children and adolescents with hearing loss (HL) face additional challenges in these transitions, which can affect their social and academic inclusion^(2)^. In this scenario, speech-language pathology support is fundamental to promoting communicative, social, and emotional skills, as well as to providing continuous support to families, thereby favoring adherence to the therapeutic process^(3)^.

Despite the importance of this support, interactive resources for this audience remain scarce in Brazil. The Growing Up with Hearing Loss material, developed by the Ida Institute, was created to empower children, adolescents, and young adults with hearing loss to face major life transitions more thoroughly prepared. The tool is organized into modules by age group (0-3 years, 3-6 years, 6-12 years, 12-18 years, and 18+ years) and includes specific content for family members and hearing healthcare professionals.

Each module is structured around three main topics: Be Inspired by Others (videos featuring testimonials of real experiences), Am I Ready? (reflective questionnaires to identify needs and strategies) and Develop New Skills (practical activities that provoke the development of social, communicative, emotional, cognitive, self-regulation, and self-determination skills).

With this format, the tool encourages individual and collective reflection, open discussion about hearing loss, strengthening family relationships, and building self-advocacy. For professionals, it offers a structured set of digital resources that support clinical practice and promote greater adherence to treatment.

During the COVID-19 pandemic, the use of digital platforms such as YouTube, Moodle, Facebook, e-Class, e-Me, Webex, and Webinar showed great potential to expand remote access to audiological and educational interventions, highlighting the relevance of tools such as the one analyzed in this study^(4)^.

Given the scarcity of materials adapted to Brazilian Portuguese and the importance of strategies that strengthen the auditory rehabilitation process, this study aims to present the localization and validation process of Growing Up with Hearing Loss into Brazilian Portuguese. The proposal seeks to broaden its applicability in Brazil and contribute to the comprehensive development of individuals with hearing loss throughout different phases of life.

## METHOD

This is a methodological study of translation, cultural adaptation, and content validation, approved by the Research Ethics Committee of FOB-USP (CAAE: 22179019.1.0000.5417; Opinion No. 5.471.523). The copyright holder of the material, Growing Up with Hearing Loss (Ida Institute), granted formal authorization for its localization and validation into the Brazilian context. The translation of the material was made possible through an academic cooperation agreement signed between the Ida Institute and FOB-USP (Process No. 17.1.4674.25.0) with the objective of contributing to auditory habilitation and rehabilitation processes, audiological education and research, promoting advances in hearing care and in the humanistic training of the campus. All participants signed the Informed Consent Form in accordance with the guidelines of CNS Resolution 466/12.

### Localization steps

Localization is the process of linguistic and cultural adaptation of material for a specific target audience, considering not only language but also sociocultural, economic, and legal aspects of the target population^(5,6)^. Following widely recognized guidelines for cross-cultural adaptation and validation of educational content in health^(5-7)^, the process adopted in this study comprised translation, cultural adaptation, and expert review^(8)^. The translator, along with other professionals involved, considered these specificities to ensure that the localized version met the needs of the target audience.

The translation and adaptation of the material occurred before any applications to participants. Subsequently, a technical review of the translated text was carried out. The steps followed adapted methodological guidelines, prioritizing semantic and cultural equivalence between the languages^(8)^. Two bilingual translators, fluent in English and Brazilian Portuguese, carried out the initial translation, applying techniques of transposition, modulation, and cultural adaptation^(9)^ to naturalize the content to the Brazilian audience.

The back-translation step was not carried out due to the material's multimodal nature (videos, questionnaires, and activities), and emphasis was placed on cultural validation through expert review, as recommended for complex educational materials^(5-7)^.

During the translation process, different procedures were used according to the nature of the divergences found. In cases of linguistic and stylistic convergence, literal or word-for-word translation was used. When linguistic divergences were identified, transposition, modulation, and equivalence techniques were applied. Stylistic divergences were addressed through omission, explication, compensation, sentence reconstruction, and formal adjustments. Extralinguistic divergences were resolved through transfer, explanation, calque, and adaptation, ensuring semantic and cultural adequacy for the Brazilian target audience^(9)^.

After the translation, three experienced speech-language pathologists (with an average of 9 years of experience; 2 with experience in auditory rehabilitation and 1 in public health) reviewed the material, analyzing its clarity, relevance, and clinical applicability. They were selected based on proven expertise and evaluated the texts for necessary linguistic and cultural adjustments.

In addition to the texts, the videos in the material were transcribed, translated, and subtitled, while respecting Brazil's cultural and linguistic aspects. Due to restrictions imposed by the COVID-19 pandemic, the original recordings were maintained, with the addition of subtitles in Brazilian Portuguese. The layout organized texts, videos, and practical activities on an online testing platform.

The Growing Up with Hearing Loss tool includes modules targeted at different age groups (children, adolescents, young adults, and their families) and a specific module for speech-language pathologists. Each module includes videos with testimonials, practical activities, and reflective questionnaires. Each group of participants exclusively evaluated the modules corresponding to their age group, ensuring the suitability of the validated content for the specific target audience.

### Validation steps

The validation process included 42 participants, distributed in four groups: G1 – children and adolescents with HL (*n* = 7; ages 9-17 years, users of electronic devices), G2 – young adults with HL (*n* = 7; ages 18-29 years), G3 – parents or guardians of children with HL (*n* = 12; children up to 8 years and 11 months), and G4 – speech-language pathologists (*n* = 18) working in auditory habilitation and rehabilitation. Participants were selected in person at FOB-USP and through social media outreach. Data collection was performed remotely via Google Forms®, with access to translated material and questionnaires specific to each group. Inclusion criteria: use of hearing aids, oral communication skills, and access to digital technologies (computer or smartphone with internet) for G1–G3; and proven clinical experience in auditory habilitation/rehabilitation for G4.

### Questionnaires used

For the validation step, three questionnaire models were used, developed according to participants’ profile. For children and adolescents (G1), a Simplified Questionnaire (Q1) was used, consisting of objective questions with the following response options: “Yes”, “No”, and “I don't know”. For young adults with HL and their parents/guardians (G2 and G3), the Descriptive Questionnaire (Q2) was used, which contains open-ended questions, agreement scales, and room for additional comments. For speech-language pathologists (G4), the Educational Validation in Health Instrument (IVCES)^(10)^ was used, structured on an ordinal scale from 0 to 2 points. The qualitative responses were examined through categorical content analysis, which identified recurring themes related to caption clarity, cultural appropriateness, and the need for audiovisual adjustments.

### Data analysis

Data analysis was performed using SPSS® version 25 software (IBM Corp., Armonk, NY, USA)^(11)^. Initially, a descriptive analysis was carried out to characterize the groups, using means, standard deviations, and frequencies. The internal consistency of the instruments was verified using Cronbach’s Alpha coefficient, considering acceptable values ​​between 0.70 and 0.90^(12)^. The agreement of responses was calculated using simple agreement, adopting cut-off points of ≥75% for the user groups and ≥80% for the speech-language pathologists^(10-13)^. The Content Validity Index (CVI) was not applied due to the tool's multimodal and qualitative nature, and a percentage agreement analysis was used, following the DISABKIDS/WHO Guidelines^(7)^.

## RESULTS

The results of this study were organized into two main stages: the localization of the materials and the validation of the translated materials.

### Localization of materials

The first stage consisted of translating and culturally adapting the counseling materials from Growing Up with Hearing Loss, developed by the Ida Institute. This process included not only literal translation but also necessary cultural adjustments to ensure the material's accessibility and relevance to the Brazilian audience. As part of this stage, 32 videos were subtitled in Portuguese, considering cultural and contextual specificities.

The material was analyzed by speech-language pathologists with clinical experience, who provided important suggestions for adapting it to the local context. Among the contributions, the following stood out: the replacement of technical terms with expressions more accessible to the lay public; the inclusion of specific Brazilian cultural examples—such as family situations in public schools—; the recommendation to add videos with testimonials from Brazilians with hearing loss, which favors user identification with the content; and suggestions for improvements in the tool’s design and navigation, in order to make it more intuitive and attractive.

**Figure 1:**
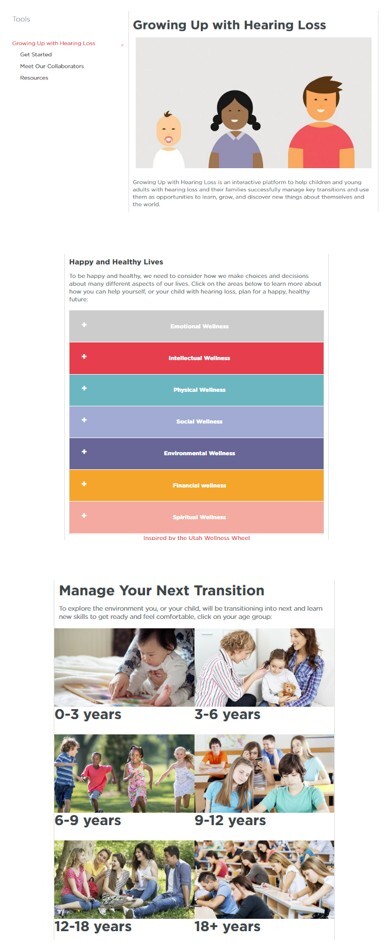
This is a Brazilian Portuguese version of the Growing Up with Hearing Loss material, titled Crescendo com a Perda Auditiva. The interface features modules on well being and life transitions for individuals with hearing loss^(14)^

These contributions were added to the test version of the material, which was subsequently converted to PDF. The final version was titled “Growing Up with Hearing Loss”, adapted to meet the needs of the Brazilian target audience (Figure 1). The material is available in open access in the University of São Paulo (USP) Repository^(14)^.

### Validation of translated materials

**Figure 2:**
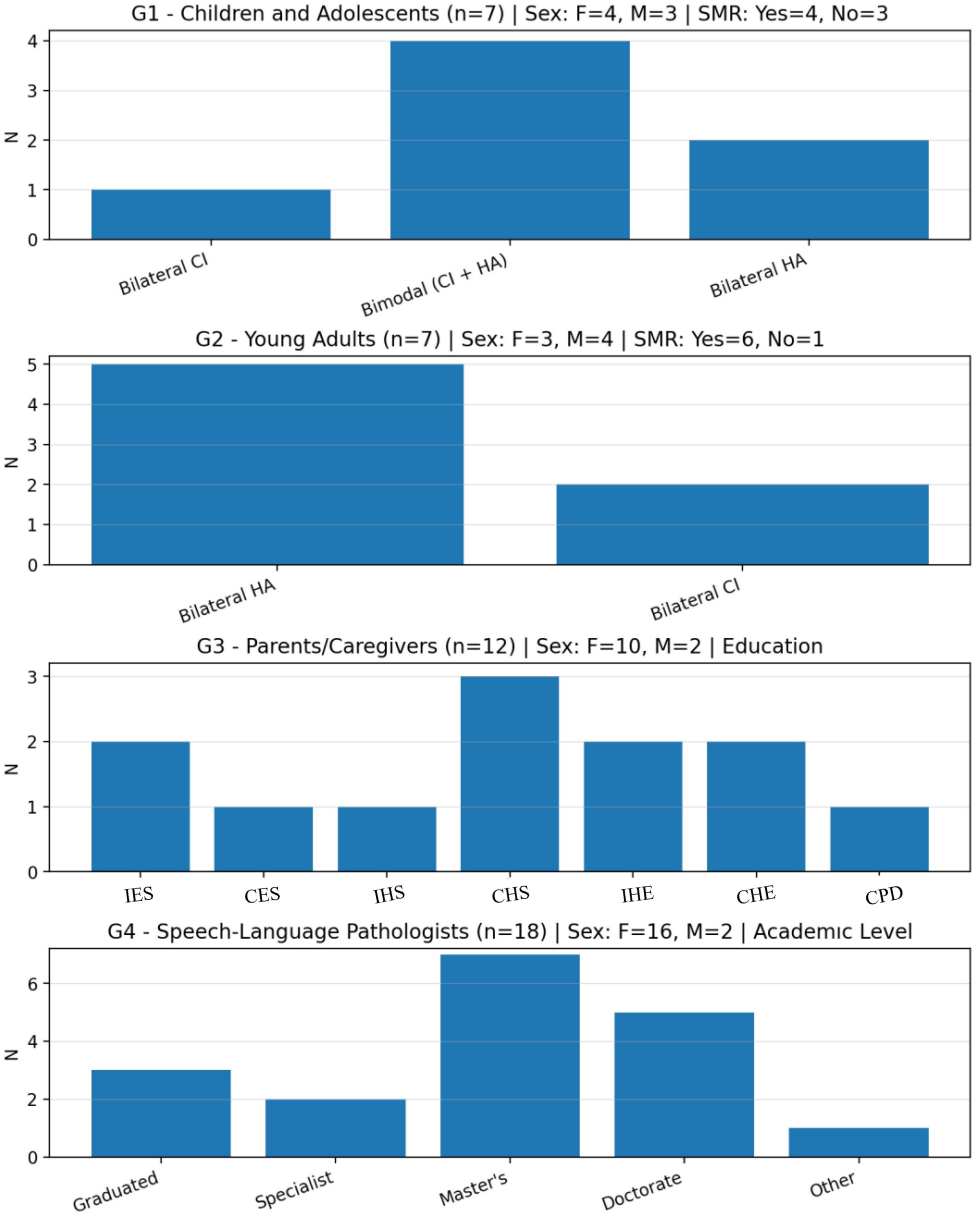
Distribution of participants according to demographic characteristics of the four study groups

The validation was performed with four different groups: children and adolescents with HL (G1A), young adults with HL (G1B), parents or guardians (G1C), and speech-language pathologists (G2). Figure 2 illustrates the demographic profile of the participants in the four study groups. The results are presented descriptively, without statistical comparisons between the groups.

Caption: G1 = Children and adolescents with hearing loss (HL), characterized by gender, use of a remote microphone system (RMS) and amplification device (hearing aid - HA; cochlear implant - CI; bimodal: CI associated with hearing aid); G2 = Young adults with HL, characterized by gender, use of RMS and amplification device; G3 = Parents or guardians, characterized by gender and education level (IES: Incomplete Elementary School; CES: Complete Elementary School; IHS: Incomplete High School; CHS: Complete High School; IHE: Incomplete Higher Education; CHE: Complete Higher Education; CPD: Complete Postgraduate Degree); G4 = Speech-language pathologists, characterized by gender and academic level (Bachelor; Specialist; Master; Ph.D.; Other)

#### Children and adolescents (G1A)

In this group, the agreement rate was 76.5%, meeting the minimum established criterion (≥75%). Participants positively evaluated the importance of the material and the simple language used. The main limitation identified was the difficulty in reading the video subtitles. Cronbach’s Alpha coefficient was negative (−1.15), likely due to the reduced number of participants and items in the tool.

#### Young adults (G1B)

The agreement rate was 74%, slightly below the cutoff point. Items related to clarity of information and promotion of personal reflection were well received. The main complaints concerned the need for more detail on topics such as cochlear implants and forms of interaction, according to age group. Cronbach’s Alpha was 0.89, indicating high internal consistency.

#### Parents and guardians (G1C)

In this group, the agreement rate was 83.3%, exceeding the established minimum criterion. Participants recognized the clarity and usefulness of the material for family support. As a suggestion, they highlighted the interest in content more applicable to daily routines and practical needs. Cronbach’s Alpha was 0.90, also indicating high internal consistency.

#### Speech-language pathologists (G2)

This group had the highest agreement rate (92.9%) and a Cronbach’s Alpha of 0.85, indicating good internal consistency. The professionals highlighted the tool’s relevance, praising the suitability of the written and visual language and emphasizing the videos as a differentiating factor for clinical practice. However, some criticisms were raised, such as the length of the texts, which was considered demotivating for adolescents, and the unattractive navigability. Among the suggestions for improvement, the need for more practical, intuitive access to the tool's steps, the inclusion of Portuguese-language videos featuring testimonials from Brazilians with HL, and the addition of more interactive graphic elements stand out.

The participants’ qualitative comments, including praise, criticism, and suggestions for improvement, are presented in Chart 1.

**Chart 1:** Participants feedback on the Growing Up with Hearing Loss tool

**Participants**	**Feedback on the tool: criticisms, praise, or suggestions related to the information, text, language, questionnaires, and videos.**			
	**Praise**	**Criticisms**	**Suggestions**	
Young adults with hearing loss	“(...) were able to understand what was written, the texts went beyond my perspective (...)”.	———-	———-	
	“I found it necessary and it helped me reflect a little better on my needs”.			
	“The questionnaires and videos brought me a lot of reflection regarding everything I've been through from childhood until today, at 22 years old (...)”.			
	“This level of detail is very helpful in presenting information about hearing loss”.			
Parents and/or guardians of children with hearing impairments	“I found it quite useful and important”.	———-	“Further explain about cochlear implants”.	
			“I would like to learn more practical ways of interacting by age group”.	
**Participants**	**Feedback on the tool: criticisms, praise, or suggestions related to the information, text, language, questionnaires, and videos.**			
	**Praise**	**Criticisms**	**Suggestions**	
Speech-language pathologists	“The tool is very relevant, the written and visual language is very well applied, and the videos act as an important complement”.	“I found the amount of text to be quite long, which could end up demotivating teenagers (...)”.	“Access to the stages needs to be faster and more intuitive. It needs to look “modern”, with more attractive visual language and more dynamic access to specific content”.	
	“A very clear and illustrative tool on the subject. I emphasize that the videos are a differentiating factor for the work”.	“I found the tool difficult to use. It’s not attractive as it is!”	“The videos could be in Portuguese, featuring intervention experiences with native Portuguese speakers. I feel the graphic material could be more interesting, attractive, intuitive, and interactive”.	
	“Excellent content and suitable for its purpose (...)”.	“The texts are relatively long”.	———-	
**Participants**	**Feedback on the tool: criticisms, praise, or suggestions related to the information, text, language, questionnaires, and videos.**			
	**Praise**	**Criticisms**	**Suggestions**	
Speech-language pathologists	“I loved how the videos are related to and illustrate the texts”.		“There’s a lot of text”.	———-
	“(...) It helps the professional to understand aspects of the family that are not accessible in a simple conversation or counseling session, it “provokes” reflection from both the family and the professional (...) The texts are written in accessible language. The proposed questionnaires are excellent (...)”.			
	“The tool is very necessary; I believe that the testimonial videos will make the people who access it engage with the tool (...) Texts with necessary information”.		———-	———-

## DISCUSSION

The main contribution of this study was the translation, cultural adaptation, and initial validation of the **Growing Up with Hearing Loss** tool into Brazilian Portuguese, resulting in the Crescendo com a Perda Auditiva version. This is a pioneering initiative in the national context, expanding access to culturally adapted hearing counseling resources aligned with person-centered care. This achievement represents an important advance for speech-language pathology clinical practice in Brazil, as it promotes adherence to treatment and strengthens the protagonism of users and their families in the rehabilitation process.

The analysis of the results showed high agreement among the three participating groups, especially among children and adolescents, who positively evaluated the language and textual content. This finding is particularly relevant and unexpected, given the difficulties described in the literature with this audience's reading of subtitles^(15,16)^. On the other hand, the group of young adults presented an agreement rate of 74%, slightly below the established minimum criterion. This result deserves attention, as it may reflect factors such as text length, linguistic complexity, or a lack of more in-depth cultural adaptation in the audiovisual materials. These hypotheses suggest the need for specific adjustments for this audience, including more attractive, interactive content contextualized to the Brazilian reality.

The participants’ suggestions reinforced these findings, indicating the importance of simplifying technical terms, including practical examples linked to Brazilian daily life, and expanding accessibility resources, such as dubbing and sign language interpreters, to promote comprehension, especially among children and adolescents. Parents and guardians also emphasized the relevance of integrating content on psychosocial aspects into the material, such as self-esteem and coping with bullying, which reinforces the role of hearing counseling not only in communicative rehabilitation but also in providing emotional support for individuals with hearing loss.

From a practical standpoint, the results suggest that the tool can be incorporated as a complementary resource to speech therapy sessions, allowing patients and families to reflect on their experiences and develop greater autonomy in the rehabilitation process. However, its large-scale implementation may face barriers, such as unequal access to digital technologies and the limited familiarity of some professionals with these resources. These limitations indicate the need for complementary strategies, including professional training and the development of simplified printed versions for contexts with less technological access.

This study has significant limitations. The small number of children and young adults limits the statistical robustness of the findings, although it represents an initial validation step. Furthermore, the lack of more in-depth cultural adaptation in the audiovisual materials may have contributed to the reported difficulties in reading subtitles, highlighting the need to explore alternatives such as dubbing or translation into Brazilian Sign Language (LIBRAS). Additionally, convenience sampling and remote data collection may have introduced selection bias, favoring participants with greater internet access and technological familiarity.

Despite these limitations, the results are in line with the findings of other international tools from the same group, such as Living Well Online and My Turn to Talk^(17)^, and with the Brazilian study^(18)^, which also highlighted the benefits of culturally adapted materials. Thus, the present work represents a fundamental initial step toward consolidating hearing counseling resources in Brazil, with the potential to enrich clinical practice and promote greater adherence to treatment. Future versions, with complete audiovisual localization and expanded accessibility features, could maximize these benefits and overcome the identified barriers.

## CONCLUSION

The validation of the Growing Up with Hearing Loss tool demonstrated its suitability to the Brazilian context, with high agreement rates in three of the four groups evaluated and recognition of its usefulness in supporting communication and managing life transitions for individuals with hearing loss. The young adult group showed a 74% agreement rate, slightly below the established minimum criterion, and children and adolescents reported difficulties understanding the subtitles, indicating areas for improvement. These findings reinforce the relevance of cultural adaptation and accessible language for the effectiveness of the tool.
