# Supplementary material for: Localization and validation of the “Growing Up With Hearing Loss” tool for Brazilian Portuguese
Source: Codas. 2026 Apr 27;38(3):e20250187. doi: 10.1590/2317-1782/e20250187en (PMC13193192; doi:10.1590/2317-1782/e20250187en)
Supplement: Translation

# Translation: Localização e validação da ferramenta “*Growing Up With Hearing Loss*” para o português brasileiro

**DOI:** 10.1590/2317-1782/e20250187pt

**Authors:** Daniele Baptista Nery, Aline Daniela Gomes da Silva Vieira, Anderson Vinícius de Moraes Ortega, Thais Corina Said de Angelo, Adriane Lima Mortari Moret, Natália Barreto Frederigue-Lopes, Déborah Viviane Ferrari, Leila Maria Gumushian Felipini, Regina Tangerino de Souza Jacob

**Affiliations:** 1 Departamento de Fonoaudiologia, Faculdade de Odontologia de Bauru – FOB, Universidade de São Paulo – USP - Bauru (SP), Brasil.; 2 Centro Universitário Sagrado Coração – Unisagrado - Bauru (SP), Brasil.

**Keywords:** Deficiência Auditiva, Reabilitação Auditiva, Adaptação Cultural, Comunicação, Validação

## Abstract

**Objetivo:**

Apresentar o processo de localização e validação do material “Growing Up with Hearing Loss” para o português brasileiro, visando ampliar sua aplicabilidade no contexto nacional.

**Método:**

Estudo metodológico de tradução, adaptação cultural e validação de conteúdo. Após a autorização do Ida Institute, o material foi localizado para o português brasileiro, garantindo equivalências semânticas, culturais e técnicas. O processo incluiu revisão por especialistas, legendagem de 32 vídeos, adaptação cultural e elaboração de questionários aplicados a crianças, adolescentes e adultos jovens com deficiência auditiva, a seus familiares e a fonoaudiólogos.

**Resultados:**

O material foi localizado como Crescendo com a Perda Auditiva e validado por 42 participantes. Três grupos apresentaram taxas de concordância superiores a 75% quanto à clareza e adequação dos conteúdos, enquanto o grupo de adultos jovens obteve 74%, valor próximo ao critério mínimo estabelecido. As sugestões concentraram-se na melhoria da legendagem dos vídeos. O material demonstrou potencial para apoiar a gestão de transições de vida, promover habilidades comunicativas e apoiar o desenvolvimento de indivíduos com deficiência auditiva.

**Conclusão:**

Crescendo com a Perda Auditiva mostrou-se adequado ao contexto brasileiro, com potencial para favorecer a reabilitação auditiva e o desenvolvimento de habilidades sociais, cognitivas e comunicativas. Recomenda-se aprimorar os conteúdos audiovisuais para otimizar a compreensão das legendas.

## INTRODUÇÃO

A Organização Mundial da Saúde (OMS) estima que mais de 1,5 bilhão de pessoas em todo o mundo vivem com algum grau de perda auditiva, incluindo 34 milhões de crianças. A ausência de diagnóstico e de intervenção precoce pode comprometer o desenvolvimento da linguagem, afetar o desempenho escolar e causar isolamento social, especialmente na infância e na adolescência^(1)^.

Durante essas fases do desenvolvimento, as demandas comunicativas, sociais e educacionais tornam-se mais complexas, exigindo maior autonomia e adaptação a novos contextos. Crianças e adolescentes com deficiência auditiva (DA) enfrentam desafios adicionais nessas transições, o que pode comprometer sua inclusão social e acadêmica^(2)^. Nesse cenário, o acompanhamento fonoaudiológico é fundamental para promover habilidades comunicativas, sociais e emocionais, além de oferecer suporte contínuo às famílias, favorecendo a adesão ao processo terapêutico^(3)^.

Apesar da relevância desse suporte, ainda são escassos os recursos interativos voltados a esse público no Brasil. O material Growing Up with Hearing Loss, desenvolvido pelo Ida Institute, foi criado para capacitar crianças, adolescentes e jovens adultos com DA a enfrentar, de forma mais preparada, as principais transições da vida. A ferramenta organiza-se em módulos divididos por faixas etárias (0-3 anos, 3-6 anos, 6-12 anos, 12-18 anos e 18+ anos), incluindo também conteúdos específicos para familiares e profissionais da saúde auditiva.

Cada módulo é estruturado em três tópicos principais: *Be Inspired by Others* (vídeos com depoimentos de experiências reais), *Am I Ready?* (questionários reflexivos para identificar necessidades e estratégias) e *Develop New Skills* (atividades práticas que estimulam o desenvolvimento de habilidades sociais, comunicativas, emocionais, cognitivas, de autorregulação e autodeterminação).

Com esse formato, a ferramenta favorece a reflexão individual e coletiva, a discussão aberta sobre a perda auditiva, o fortalecimento das relações familiares e a construção da autodefesa ou da autoadvocacia (advocacia pessoal). Para os profissionais, oferece um conjunto estruturado de recursos digitais que apoiam a prática clínica e promovem maior adesão ao tratamento.

Durante a pandemia de COVID-19, o uso de plataformas digitais como YouTube, Moodle, Facebook, e-Class, e-Me, Webex e Webinar mostrou grande potencial para ampliar o acesso remoto a intervenções audiológicas e educativas, evidenciando a relevância de ferramentas como a analisada neste estudo^(4)^.

Diante da escassez de materiais adaptados ao português brasileiro e da importância de estratégias que fortaleçam o processo de reabilitação auditiva, este estudo tem como objetivo apresentar o processo de localização e validação do *Growing Up with Hearing Loss* para o português brasileiro. A proposta visa ampliar sua aplicabilidade no Brasil e contribuir para o desenvolvimento integral de indivíduos com DA ao longo das diferentes fases da vida.

## MÉTODO

Trata-se de um estudo metodológico de tradução, adaptação cultural e validação de conteúdo, aprovado pelo Comitê de Ética em Pesquisa da FOB-USP (CAAE: 22179019.1.0000.5417; Parecer nº 5.471.523). O titular dos direitos autorais do material *Growing Up with Hearing Loss* (Ida Institute) concedeu autorização formal para sua localização e validação no contexto brasileiro. A tradução do material foi viabilizada por meio de um termo de cooperação acadêmica firmado entre o Ida Institute e a FOB-USP (Processo nº 17.1.4674.25.0), cujo objetivo é contribuir para os processos de habilitação e reabilitação auditivas, para a educação e a pesquisa audiológicas, promovendo avanços no cuidado da audição e na formação humanista do campus. Todos os participantes assinaram o Termo de Consentimento Livre e Esclarecido, conforme as diretrizes da Resolução CNS 466/12.

### Etapas da localização

A localização é definida como o processo de adaptação linguística e cultural de um material para um público-alvo específico, considerando não apenas o idioma, mas também aspectos socioculturais, econômicos e legais da população-alvo^(5,6)^. Seguindo diretrizes amplamente reconhecidas para a adaptação transcultural e a validação de conteúdos educacionais em saúde^(5-7)^, o processo adotado neste estudo compreendeu tradução, adaptação cultural e revisão especializada^(8)^. O tradutor, juntamente com outros profissionais envolvidos, considerou essas especificidades para assegurar que a versão localizada atendesse às necessidades do público-alvo.

A tradução e adaptação do material ocorreram antes de qualquer aplicação com participantes. Posteriormente, realizou-se uma revisão técnica do texto traduzido. As etapas seguiram diretrizes metodológicas adaptadas, priorizando equivalência semântica e cultural entre os idiomas^(8)^. Dois tradutores bilíngues, fluentes em inglês e português brasileiro, realizaram a tradução inicial, aplicando técnicas de transposição, modulação e adaptação cultural^(9)^ para naturalizar o conteúdo ao público brasileiro.

A etapa de retrotradução não foi realizada devido à natureza multimodal do material (composto por vídeos, questionários e atividades), e a ênfase foi dada à validação cultural por meio de revisão por especialistas, conforme recomendado para materiais educacionais complexos^(5-7)^.

Durante o processo de tradução, foram empregados procedimentos distintos conforme a natureza das divergências encontradas. Nos casos de convergência linguística e estilística, utilizou-se a tradução literal ou palavra por palavra. Quando identificadas divergências linguísticas, aplicaram-se técnicas de transposição, modulação e equivalência. As divergências estilísticas foram tratadas por meio de omissão, explicitação, compensação, reconstrução de períodos e ajustes formais. Já as divergências extralinguísticas foram solucionadas por meio de transferência, explicação, decalque e adaptação, assegurando a adequação semântica e cultural ao público-alvo brasileiro^(9)^.

Após a tradução, três fonoaudiólogos experientes (tempo médio de atuação: 9 anos; dois com experiência em reabilitação auditiva e um em saúde coletiva) revisaram o material, analisando a clareza, a relevância e a aplicabilidade clínica. Eles foram selecionados com base em expertise comprovada e avaliaram os textos para ajustes linguísticos e culturais necessários.

Além dos textos, os vídeos do material foram transcritos, traduzidos e legendados, respeitando os aspectos culturais e linguísticos do Brasil. Devido às restrições impostas pela pandemia de COVID-19, as gravações originais foram mantidas, com inserção de legendas em português brasileiro. A diagramação organizou textos, vídeos e atividades práticas em uma plataforma de teste online.

A ferramenta *Growing Up with Hearing Loss* é composta por módulos direcionados a diferentes faixas etárias (crianças, adolescentes, jovens adultos e seus familiares) e por um módulo específico para fonoaudiólogos. Cada módulo inclui vídeos com depoimentos, atividades práticas e questionários reflexivos. Cada grupo de participantes avaliou exclusivamente os módulos correspondentes à sua faixa etária, garantindo a adequação do conteúdo validado ao público-alvo específico.

### Etapas para validação

O processo de validação incluiu 42 participantes, organizados em quatro grupos: G1 – crianças e adolescentes com DA (n=7; idades entre 9 e 17 anos, usuários de dispositivos eletrônicos), G2 – adultos jovens com DA (n=7; idades entre 18 e 29 anos), G3 – pais ou responsáveis por crianças com DA (n=12; crianças até 8 anos e 11 meses) e G4 – fonoaudiólogos (n=18) atuantes em habilitação e reabilitação auditiva. Os participantes foram recrutados presencialmente na FOB-USP e por meio de divulgação nas mídias sociais. A coleta foi realizada remotamente, via Google Forms®, com acesso ao material traduzido e aos questionários específicos para cada grupo. Os critérios de inclusão foram: uso de dispositivos aplicativos para surdez, habilidade comunicativa oral e acesso a tecnologias digitais (computador ou smartphone com internet) para G1–G3; e experiência clínica comprovada em habilitação/reabilitação auditiva para os participantes do G4.

### Questionários utilizados

Para a etapa de validação, foram utilizados três modelos de questionários, elaborados de acordo com o perfil dos participantes. Para crianças e adolescentes (G1) aplicou-se um Questionário Simplificado (Q1), composto por perguntas objetivas com opções de resposta “Sim”, “Não” e “Não sei”. Para adultos jovens com DA e pais/responsáveis (G2 e G3), utilizou-se o Questionário Descritivo (Q2), que contém questões abertas, escalas de concordância e espaço para comentários adicionais. Já para os fonoaudiólogos (G4) foi aplicado o Instrumento de Validação Educativa em Saúde (IVCES)^(10)^, estruturado em escala ordinal de 0 a 2 pontos. As respostas qualitativas foram examinadas por meio de análise de conteúdo categorial, identificando temas recorrentes relacionados à clareza das legendas, à adequação cultural e à necessidade de ajustes audiovisuais.

### Análise de dados

A análise dos dados foi realizada no software SPSS® versão 25 (IBM Corp., Armonk, NY, EUA)^(11)^. Inicialmente, foi conduzida uma análise descritiva para caracterização dos grupos, utilizando médias, desvios-padrão e frequências. A consistência interna dos instrumentos foi verificada por meio do coeficiente Alfa de Cronbach, considerando-se valores aceitáveis entre 0,70 e 0,90^(12)^. A concordância das respostas foi calculada por meio de concordância simples, adotando-se como pontos de corte ≥75% para os grupos de usuários e ≥80% para os fonoaudiólogos^(10-13)^. O Índice de Validade de Conteúdo (IVC) não foi aplicado devido ao caráter multimodal e qualitativo da ferramenta, optando-se pela análise de concordância percentual, conforme as orientações das DISABKIDS/WHO Guidelines^(7)^.

## RESULTADOS

Os resultados deste estudo foram organizados em duas etapas principais: a localização dos materiais e a validação dos materiais traduzidos.

### Localização dos materiais

A primeira etapa consistiu na tradução e na adaptação cultural dos materiais de aconselhamento do *Growing Up with Hearing Loss*, desenvolvidos pelo Ida Institute. Esse processo incluiu não apenas a tradução literal, mas também ajustes culturais necessários para assegurar a acessibilidade e a relevância do material para o público brasileiro. Como parte dessa etapa, 32 vídeos foram legendados em português, considerando as especificidades culturais e contextuais.

O material foi analisado por fonoaudiólogos com experiência clínica, que forneceram sugestões importantes para a adequação ao contexto nacional. Entre as contribuições, destacaram-se a substituição de termos técnicos por expressões mais acessíveis ao público leigo, a inclusão de exemplos culturais específicos do Brasil — como situações familiares em escolas públicas —, a recomendação de incorporar vídeos com depoimentos de brasileiros com DA, o que favorece a identificação dos usuários com o conteúdo, além de sugestões de melhorias no design e na navegação da ferramenta, de modo a torná-la mais intuitiva e atrativa.

Essas contribuições foram incorporadas à versão de teste do material, que posteriormente foi convertida para PDF. A versão final recebeu o título “Crescendo com perda auditiva”, adaptada para atender às necessidades do público-alvo brasileiro (Figura 1). O material está disponível em acesso aberto no Repositório da Universidade de São Paulo (USP)^(14)^.

**Figura 1:** Versão localizada no material *Growing Up with Hearing Loss* para protuguês brasileiro, intitulada *Crescendo com a Perda Auditiva*. A interface apresenta módulos sobre bem-estar e transições ao longo da vida de indivíduos com deficiência auditiva^(14)^

### Validação dos materiais traduzidos

A validação foi conduzida com quatro grupos distintos: crianças e adolescentes com DA (G1A), adultos jovens com DA (G1B), pais ou responsáveis (G1C) e fonoaudiólogos (G2). A Figura 2 ilustra o perfil demográfico dos participantes dos quatro grupos do estudo. Os resultados são apresentados de forma descritiva, sem comparações estatísticas entre os grupos.

**Figura 2:** Distribuição dos participantes segundo características demográficas dos quatro grupos do estudo

Legenda: G1 = Crianças e adolescentes com deficiência auditiva (DA), caracterizados por sexo, uso de sistema de microfone remoto (SMR) e dispositivo de amplificação (aparelho de amplificação sonora individual – AASI; implante coclear – IC; bimodal: IC associado a AASI); G2 = Adultos jovens com DA, caracterizados por sexo, uso de SMR e dispositivo de amplificação; G3 = Pais ou responsáveis, caracterizados por sexo e escolaridade (EFI: Ensino Fundamental Incompleto; EFC: Ensino Fundamental Completo; EMI: Ensino Médio Incompleto; EMC: Ensino Médio Completo; ESI: Ensino Superior Incompleto; ESC: Ensino Superior Completo; PGC: Pós-Graduação Completa); G4 = Fonoaudiólogos, caracterizados por sexo e nível acadêmico (Graduado; Especialista; Mestre; Doutor; Outro)

#### Crianças e adolescentes (G1A)

O grupo apresentou taxa de concordância de 76,5%, atendendo ao critério mínimo estabelecido (≥75%). Os participantes avaliaram positivamente a importância do material e a linguagem simples empregada. A principal limitação identificada foi a dificuldade de leitura das legendas dos vídeos. O coeficiente Alfa de Cronbach apresentou valor negativo (-1,15), atribuído ao número reduzido de participantes e de itens do instrumento.

#### Adultos jovens (G1B)

A taxa de concordância foi de 74%, ligeiramente abaixo do ponto de corte. Os itens relacionados à clareza das informações e à promoção de reflexões pessoais tiveram boa aceitação. As principais críticas referiram-se à necessidade de maior detalhamento sobre temas como o implante coclear e as formas de interação, de acordo com a faixa etária. O Alfa de Cronbach foi de 0,89, indicando alta consistência interna.

#### Pais e responsáveis (G1C)

Nesse grupo, a taxa de concordância foi de 83,3%, superando o critério mínimo estabelecido. Os participantes reconheceram a clareza e a utilidade do material para o apoio familiar. Como sugestão, destacaram o interesse por conteúdos mais aplicáveis à rotina e às necessidades práticas. O Alfa de Cronbach foi de 0,90, também indicando alta consistência interna.

#### Fonoaudiólogos (G2)

Este grupo apresentou a maior taxa de concordância (92,9%), com Alfa de Cronbach de 0,85, o que representa boa consistência interna. Os profissionais destacaram a relevância da ferramenta, elogiando a adequação da linguagem escrita e visual e ressaltando os vídeos como um diferencial para a prática clínica. Entretanto, algumas críticas foram apontadas, como a extensão dos textos, considerada desmotivadora para adolescentes, e a navegabilidade pouco atraente. Entre as sugestões de aprimoramento, destacam-se a necessidade de um acesso mais prático e intuitivo às etapas da ferramenta, a inclusão de vídeos em português com vivências de brasileiros com DA e a incorporação de elementos gráficos mais interativos.

Os comentários qualitativos dos participantes, incluindo elogios, críticas e sugestões de aprimoramento, são apresentados no Quadro 1.

**Quadro 1:** Comentários dos participantes sobre a ferramenta Crescendo com a perda auditiva

**Participantes**	**Opinião sobre a ferramenta: críticas, elogios ou sugestões relacionadas às informações, texto, linguagem, questionários e vídeos.**			
	**Elogios**	**Críticas**	**Sugestões**	
Adultos jovens com deficiência auditiva	“(...) deu para entender o que estava escrito, os textos foram além da minha perspectiva (...)”.	----------	----------	
	“Achei necessário e me ajudou a refletir um pouco melhor nas minhas necessidades”.			
	“Os questionários e vídeos me trouxeram muitas reflexões em relação ao tudo o que passei desde a infância até hoje com 22 anos (...).”			
	“Esse detalhe é muito bom para mostrar as informações de perdas auditivas”.			
Pais e/ou responsáveis por crianças com deficiência auditiva	"Achei bastante útil e importante".	----------	“Explicar mais sobre o implante coclear”	
			“Gostaria de aprender formas mais práticas de interação por idade”.	
Fonoaudiólogos	“A ferramenta é muito relevante, a linguagem escrita e visual está muito bem aplicada, e os vídeos atuam como um complemento importante”.	“Achei a quantidade de textos grandes, o que pode acabar desmotivando adolescentes (...)”.	“O acesso às etapas precisaria ser mais rápido e intuitivo. Precisa parecer "moderna", com linguagem visual mais atrativa e acesso aos conteúdos específicos mais dinâmicos”.	
	“Ferramenta bem clara e ilustrativa sobre o tema. Ressalto que os vídeos são um diferencial no trabalho”.	“Achei difícil manusear a ferramenta. Da forma como está não é atraente!”	“Os vídeos poderiam ser em português, com vivências de intervenção com nativos da língua portuguesa. Sinto falta de um material gráfico mais interessante, atrativo, intuitivo e interativo”.	
	“Trabalho de excelente conteúdo e adequado ao que se propõe (...)”.	“Os textos são relativamente longos”.	----------	
Fonoaudiólogos	“Adorei a forma como os vídeos estão relacionados e ilustrando os textos”.		“Tem bastante texto”.	----------
	“(...) Auxilia o profissional a conhecer aspectos da família que não se tem acesso numa simples conversa ou aconselhamento, "provoca" reflexão por parte da família e do profissional (...) Os textos são construídos com linguagem acessível. Os questionários propostos estão ótimos (...).			
	“Muito necessária a ferramenta, acredito que os vídeos dos depoimentos farão com que as pessoas que acessarem se envolvam com a ferramenta (...) Textos com informações necessárias”.		----------	----------

## DISCUSSÃO

O presente estudo teve como principal contribuição a tradução, a adaptação cultural e a validação inicial da ferramenta ***Growing Up with Hearing Loss*** para o português brasileiro, resultando na versão **Crescendo com a Perda Auditiva**. Trata-se de uma iniciativa inédita no contexto nacional, que amplia o acesso a recursos de aconselhamento auditivo culturalmente adaptados e alinhados ao cuidado centrado na pessoa. Esse mérito representa um avanço importante para a prática clínica fonoaudiológica no Brasil, pois favorece a adesão ao tratamento e fortalece o protagonismo dos usuários e familiares no processo de reabilitação.

A análise dos resultados evidenciou alta concordância entre os três grupos participantes, especialmente entre crianças e adolescentes, que avaliaram positivamente a linguagem e o conteúdo textual. Esse achado é particularmente relevante e até inesperado, considerando as dificuldades descritas na literatura quanto à leitura de legendas por esse público^(15,16)^. Por outro lado, o grupo de jovens adultos apresentou taxa de concordância de 74%, ligeiramente abaixo do critério mínimo estabelecido. Esse resultado merece atenção, pois pode refletir fatores como a extensão dos textos, o nível de complexidade linguística ou ainda a falta de uma adaptação cultural mais aprofundada nos materiais audiovisuais. Tais hipóteses sugerem a necessidade de ajustes específicos para esse público, incluindo conteúdos mais atrativos, interativos e contextualizados à realidade brasileira.

As sugestões dos participantes reforçaram esses achados, indicando a importância de simplificar termos técnicos, incluir exemplos práticos vinculados ao cotidiano brasileiro e ampliar os recursos de acessibilidade, como dublagem e intérpretes de LIBRAS, para favorecer a compreensão, especialmente entre crianças e adolescentes. Pais e responsáveis ainda ressaltaram a relevância de integrar ao material conteúdos relacionados a aspectos psicossociais, como a autoestima e o enfrentamento do bullying, o que reforça o papel do aconselhamento auditivo não apenas na reabilitação comunicativa, mas também no suporte emocional aos indivíduos com DA.

Do ponto de vista prático, os resultados sugerem que a ferramenta pode ser incorporada como recurso complementar às sessões fonoaudiológicas, permitindo que pacientes e familiares reflitam sobre suas experiências e desenvolvam maior autonomia no processo de reabilitação. Contudo, sua implementação em larga escala pode enfrentar barreiras, como o acesso desigual às tecnologias digitais e a familiaridade limitada de alguns profissionais com esses recursos. Tais limitações indicam a necessidade de estratégias complementares, incluindo capacitação profissional e o desenvolvimento de versões simplificadas em formato impresso para contextos de menor acesso tecnológico.

Este estudo também apresenta limitações relevantes. O número reduzido de crianças e jovens adultos limita a robustez estatística dos achados, embora represente uma etapa inicial de validação. Além disso, a ausência de uma adaptação cultural mais aprofundada nos materiais audiovisuais pode ter contribuído para as dificuldades relatadas na leitura de legendas, o que aponta para a necessidade de explorar alternativas, como a dublagem ou a tradução em LIBRAS. Ademais, a amostragem por conveniência e a coleta remota podem ter introduzido viés de seleção, favorecendo participantes com maior acesso à internet e maior familiaridade tecnológica.

Apesar dessas limitações, os resultados alinham-se aos achados de outras ferramentas internacionais do mesmo grupo, como *Living Well Online* e *My Turn to Talk* ^(17)^, e ao estudo brasileiro^(18)^, que também destacou os benefícios de materiais culturalmente adaptados. Assim, o presente trabalho representa um passo inicial fundamental para a consolidação de recursos de aconselhamento auditivo no Brasil, com potencial para enriquecer a prática clínica e promover maior adesão ao tratamento. Versões futuras, com localização audiovisual completa e recursos de acessibilidade ampliados, poderão maximizar esses benefícios e superar as barreiras identificadas.

## CONCLUSÃO

A validação da ferramenta Crescendo com a Perda Auditiva evidenciou sua adequação ao contexto brasileiro, com altas taxas de concordância em três dos quatro grupos avaliados e o reconhecimento de sua utilidade no apoio à comunicação e no gerenciamento das transições de vida de indivíduos com DA. O grupo de jovens adultos apresentou taxa de concordância de 74%, ligeiramente abaixo do critério mínimo estabelecido, e crianças e adolescentes relataram dificuldades na compreensão das legendas, apontando aspectos que demandam aprimoramento. Esses achados reforçam a relevância da adaptação cultural e da linguagem acessível para a efetividade da ferramenta.
